# *Pseudomonas aeruginosa* Keratitis: Protease IV and PASP as Corneal Virulence Mediators

**DOI:** 10.3390/microorganisms7090281

**Published:** 2019-08-22

**Authors:** Richard O’Callaghan, Armando Caballero, Aihua Tang, Michael Bierdeman

**Affiliations:** Department of Microbiology and Immunology, University of Mississippi Medical Center, Jackson, MS 39216, USA

**Keywords:** *Pseudomonas aeruginosa*, bacterial keratitis, corneal virulence, protease IV (PIV), *P. aeruginosa* small protease (PASP), catalytic triad, corneal ulcer, respiratory infection, insect infection, plant infection

## Abstract

*Pseudomonas aeruginosa* is a leading cause of bacterial keratitis, especially in users of contact lenses. These infections are characterized by extensive degradation of the corneal tissue mediated by *Pseudomonas* protease activities, including both *Pseudomonas* protease IV (PIV) and the *P. aeruginosa* small protease (PASP). The virulence role of PIV was determined by the reduced virulence of a PIV-deficient mutant relative to its parent strain and the mutant after genetic complementation (rescue). Additionally, the non-ocular pathogen *Pseudomonas putida* acquired corneal virulence when it produced active PIV from a plasmid-borne *piv* gene. The virulence of PIV is not limited to the mammalian cornea, as evidenced by its destruction of respiratory surfactant proteins and the cytokine interleukin-22 (IL-22), the key inducer of anti-bacterial peptides. Furthermore, PIV contributes to the *P. aeruginosa* infection of both insects and plants. A possible limitation of PIV is its inefficient digestion of collagens; however, PASP, in addition to cleaving multiple soluble proteins, is able to efficiently cleave collagens. A PASP-deficient mutant lacks the corneal virulence of its parent or rescue strain evidencing its contribution to corneal damage, especially epithelial erosion. *Pseudomonas*-secreted proteases contribute importantly to infections of the cornea, mammalian lung, insects, and plants.

## 1. Introduction: *Pseudomonas aeruginosa* Keratitis and the Role of the Secreted Proteases

*Pseudomonas aeruginosa* causes infections in animals ranging from mammals to insects, and in plants. The organism is found in many environments, especially those with a significant water content. Among humans, *P. aeruginosa* is responsible for community-acquired infections, as well as approximately 10% of all nosocomial infections including infections of the skin, wounds, lung, and blood [1]. The community-acquired infections include wound and other opportunistic infections, but human corneal infections have been recognized as especially harmful and difficult to treat [2]. *Pseudomonas* is a major cause of keratitis that develops primarily in association with the use (misuse) of contact lenses [3]. *P. aeruginosa* keratitis is characterized by the development of corneal ulcers and, even with proper antibiotic therapy, has the potential to develop corneal scarring that impairs vision. Cases of *Pseudomonas* keratitis present a poor prognosis, especially when therapy is not initiated early in the infection. Even when antibiotic therapy rapidly kills the infecting bacteria, the infected eye continues to be damaged by the products of the organisms and those of the inflammatory response induced by the infection [4]. Although steroids and other anti-inflammatory drugs can help limit the damaging immune response, there are no medications able to inhibit the activity of the bacterial products [5]. A key objective of research on *Pseudomonas* keratitis has been the identification of the bacterial products that damage the eye, with a long-term goal of developing means to inhibit the activity of these bacterial products. Inhibition of these bacterial pathogenic mechanisms is recognized as a means to treat infections, especially when antibiotic resistance is problematic [6].

Numerous efforts have been made to understand the molecular processes that result in the devastating corneal damage that can occur in *Pseudomonas* keratitis. There are host factors associated with the inflammatory response that can mediate tissue damage. The infiltration of the corneal stroma by polymorphonuclear neutrophils (PMN) provides a source of damaging enzymes and reactive oxygen species including peroxides, superoxide, hydroxyl radical, and singlet oxygen that damage normal tissue [6]. The activation of the host matrix metalloproteinases (MMP) also contributes to the destruction of corneal tissue [7]. These host factors are probably synergistic with the multiple bacterial products that also attack the host. The ability of bacteria to survive in tissue and cause inflammation and tissue damage hinges, to a large extent, on their ability to secrete biologically active proteins [1]. 

Because *Pseudomonas* keratitis can include liquefactive necrosis of the cornea, emphasis has been given to the role of secreted bacterial proteases [8]. Proteases are thought to destroy the ground substance, resulting in the dispersal of collagen fibrils, weakening of the corneal stroma, and corneal perforation due to anterior chamber pressure. For *P. aeruginosa*, secretion of endoproteases is an important component of its virulence. *P. aeruginosa* can produce at least nine secreted endoproteases, as listed in Table 1. In 1958, a study of the *P. aeruginosa* culture supernatant showed that secreted bacterial products could cause a corneal ulcer in a rabbit eye [9]. This activity was sensitive to heat, suggesting that one or more proteins were the active component. Kawaharajo et al. found that each of two protease-producing strains of *P. aeruginosa* caused ulceration of the mouse cornea, whereas each of two non-protease producing strains failed to cause such corneal damage [10]. Purification of the secreted proteases led to identification of two metalloproteases, elastase (now elastase B, LasB) and alkaline protease [11,12,13]. In vitro studies of these proteases have shown that they can destroy cytokines [14], complement [15], immunoglobulins [16], and damage leukocytes [17]. The injection of either of these proteases into a cornea in vivo can cause significant damage. Mutants producing only about 10% of the normal protease activity were found to lack the full virulence of their parent strain; however, these mutants were produced by chemical mutagenesis, implying that multiple chromosomal sites could have been mutated [18]. Kernacki et al. also determined that the presence of alkaline protease, but not elastase B (LasB), could be detected in the infected corneal tissue at various times post-infection [19]. Complicating the study of damaging bacterial products was the discovery of a third secreted metalloprotease, elastase A (LasA) [20]. This protease, often referred to as the staphylolytic protease, is known for its ability to digest glycine-glycine bonds in the cell wall of *Staphylococcus aureus*, causing lysis of these bacteria. A mutant deficient in LasA production was found to lack the virulence of its parent strain, but subsequent studies found that the mutation removing LasA production also removed additional genes needed to achieve normal bacterial growth in the cornea [21,22,23]. Thus, the studies of the metalloproteases led to the conclusion that the metalloproteases (alkaline protease, LasA, and LasB) are not essential for initiating or maintaining a corneal infection [24].

In contrast to the unconvincing findings relative to ocular virulence and secreted metalloproteases, studies of the ocular virulence of two *Pseudomonas* serine proteases have shown their important role in such infections. The two virulence-mediating proteases are protease IV (PIV) and the *Pseudomonas aeruginosa* small protease (PASP). As described below, evidence shows that PIV also has a role in the virulence of *Pseudomonas* infections of plants, insects, and the mammalian lung, including the human lung. Furthermore, both PIV and PASP appear to be active in *Pseudomonas* lung infections of cystic fibrosis patients.

## 2. Physical, Enzymatic, and Virulence Properties of Protease IV 

### 2.1. Protease Discovery and Gene Identification

In 1986, a *P. aeruginosa* serine protease, designated as Ps-1, was identified and, because its substrate cleavage reaction was lysine-specific, its activity was recognized as being beneficial in cleaving proteins for their sequence analysis [27]. These investigators noted that Ps-1 was similar, but not identical, to endopeptidase Lys-C from *Lysobacter enzymogenes*. In 1992, mRNAs specific for the two elastases and alkaline protease could not be detected in a strain with caseinolytic activity, indicating production of an additional unknown protease [37]. The protease of this strain was not isolated but was designated as protease IV (PIV). Relative to the existence of a fourth protease, O’Callaghan et al. sought to isolate a protease from large cultures [28]. The uncharacterized PIV molecule was first isolated from culture supernatants of strain PA103-29, a strain that had been chemically mutagenized to delete exotoxin A production. In 1996, PIV was isolated and shown to produce corneal damage in a rabbit ocular model [28,38]. Native PIV was shown to migrate as aggregates on zymograms at 120 kDa, but can also form aggregates that migrate on zymograms with an apparent molecular weight of >200 kDa [39]. Migration of PIV on a casein zymogram is distinct from alkaline protease migrating as a doublet at 50 kDa and elastase B migrating at 163 kDa [39].

The N-terminal sequence of purified PIV was determined and the *piv* gene was cloned by use of phagemid cloning [40]. The N-terminal decapeptide sequence of PIV is not homologous with any published protein sequence; however, PIV is homologous to the protease found in *Achrombacter lyticus* and *Lysobacter enzymogenes* [28,38,40,41,42,43]. Active PIV has a molecular mass of 26,383.9 Daltons, as determined by mass spectrometry [38]. PCR reactions showed that the gene was found in all strains tested, but in none of the non-aeruginosa strains tested [40]. The *piv* gene is highly conserved among *P. aeruginosa* strains (i.e., 97.5% or higher similarity) [43]. PIV activity of 23 wild-type *P. aeruginosa* strains tested varied from 2.3 to 221.5 × 10^3^ U/mg protein in the culture supernatant [40]. There is a correlation between the exact sequence of the *piv* gene and the presence of the type three secretion product, that is, *exoU* or *exoS*, produced by the strain [44]. The active enzyme has been purified in its native state [38], as a recombinant protein [40], and as a recombinant that was converted back into its native state [44]. 

The *piv* gene was sequenced and the *pseudomonas* genome database (www.pseudomonas.com/) lists the gene for protease IV as *piv* and identified its chromosomal location in strain PAO1 as PA4175 [40]. Subsequently, in 2001, this protease was referred to as PrpL, a designation based on its regulation by the sigma factor (PvdS), which is also needed for pyoverdine expression [45]. However, the name “Protease IV (i.e., PIV)” will be used exclusively in this Review because it was the name assigned to it prior to its isolation and has been used commonly in publications regarding *Pseudomonas* virulence [28,38,40,41,42].

### 2.2. PIV Protein Processing 

**Figure 1:**
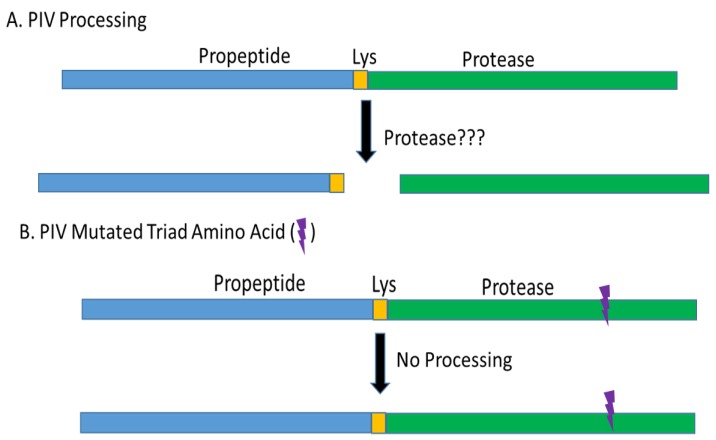
Processing of PIV. (**A**) This represents the process known to occur and emphasizes that the protease responsible for cleavage of the mature protease from the propeptide is not yet fully established. (**B**) This illustration demonstrates that a PIV molecule with a mutated catalytic residue is unable to be processed; this finding was obtained in strain PA103-29, an organism that lacks production of LasB [47].

The *piv* gene produces a 48 kDa protein with a signal sequence, a propeptide, and a mature protease domain [41]. Removing the signal sequence involves cleavage of the 48 kDa protein (i.e., pre-propeptide) to a 45 kDa protein (i.e., proenzyme) and there is subsequent cleavage of the 45 kDa proenzyme to the mature protease (~27 kDa on SDS-PAGE). The processing of the 45 kDa proenzyme to the mature protease involves removal of the propeptide region [41]. Traidej et al. found that the signal peptide was removed from the 48 kDa molecule by the time the polypeptide reached the periplasm forming the 45 kDa proenzyme. The proenzyme was then cleaved to release the mature protease found in the culture supernatant. However, when one of the amino acid residues that comprises the active site of PIV (i.e., His-72, Asp-122, or Ser-198) was mutated, the protease was inactive and only the pre-propeptide (48 kDa) and proenzyme (45 kDa) could be detected; that is, the mature protease could not be produced. These findings indicate that PIV auto-processes the proenzyme into the mature protease by cleaving the proenzyme at the lysine found at the junction between the propeptide and the mature protease. Furthermore, in the study of a non-proteolytic mutant of PIV in which an alanine was substituted for the histidine (His-72) of the catalytic triad, there was an accumulation of proenzyme and the addition of active PIV cleaved the proenzyme releasing mature PIV [41]. However, incubating heat-inactivated PIV with the proenzyme containing the alanine substitution of the histidine in the PIV catalytic triad failed to release the mature protease from the propeptide. Thus, these findings demonstrated that PIV was auto-processed by cleaving the propeptide away from the mature protease (Figure 1) [41]. However, the separation of the propeptide portion from the mature protease is not yet fully understood. Oh et al. have reported that the proenzyme is secreted into the supernatant and that LasB, but no other known *P. aeruginosa* protease, cleaves the proenzyme to activate protease IV in the supernatant [46]. Oh et al. also reported that suppression of LasB production allowed the accumulation of the proenzyme in the medium, indicating that global regulation of LasB production indirectly controlled the production of active PIV. Thus, the findings of Oh et al. differ from that of Traidej et al. and the differences in the findings appear to relate to unknown reasons or to the strains of bacteria analyzed. Traidej et al. employed strain PA103-29 and Oh et al. employed strain PAO1. A major difference in these strains is that PA103 (and its mutants including PA103-29) lacks LasB production [47]. Despite lacking LasB, PA103 strains produce considerable quantities of active PIV, indicating that LasB is not needed to produce the mature protease. Also, the *piv* gene from PA103-29 has been cloned and expressed in *E. coli* and *P. putida* and active PIV was produced by both organisms despite the absence of LasB [40,41,42]. Zhao et al. have also described the auto-activation of PIV expressed by *E. coli* [44]. Furthermore, *P. putida* KT2440 (American type culture collection) lacks detectable secreted protease activity; however, after the cloning and expression of *piv* in *P. putida,* active PIV was secreted, and the strain gained corneal virulence [42]. Thus, the production of active PIV occurs in *P. aeruginosa* PA103, *E. coli*, and *P. putida* without the action of LasB. PIV is highly conserved among numerous *P. aeruginosa* isolates with gene sequence similarity measured at 97.5% or higher and the PIV mature enzyme sequences of PA103 and PAO1 are very similar [43]. The PIV of PA103 and PAO1 do differ in four amino acids of the propeptide domain and additional differences exist in the DNA of the promoter region [40]. There is a possibility that small sequence differences in the propeptide (i.e., 4 amino acids) might explain the observed differences in the processing of PIV. 

### 2.3. Enzymatic Properties of PIV

Protease IV is not inhibited by thiol-, carboxyl-, or metalloproteinase inhibitors, but is fully inhibited by TLCK (N-p-tosyl-l-chloromethyl ketone) and partially inhibited by diisopropyl fluorophosphate or phenylmethylsulfonyl fluoride [38]. These inhibitions show that PIV is a serine protease. Inhibition of PIV activity has also been accomplished using pre-elafin [48]. Dithiothreitol or β-mercaptoethanol eliminate PIV activity, suggesting that intramolecular disulfide bonds are essential for enzyme activity [38]. As mentioned above, PIV is a serine protease and the three amino acid residues forming its catalytic triad (i.e., His-72, Asp-122, and Ser-198) are typical for a serine protease [41]. PIV has optimum enzymatic activity at pH 10.0 and 45 °C [38]. Purified PIV demonstrates activity for the carboxyl side of lysine-containing peptides and can digest a number of biologically important proteins, including immunoglobulin, complement components, fibrinogen, plasminogen, lactoferrin, transferrin, decorin, and elastin [38,40,45]. PIV can activate urokinase, leading to a cascade of proteolytic changes in the host [49]. Other important findings described below (see lung models of infection) are that PIV can cleave surfactant proteins A and D and cytokine IL-22, a cytokine able to provide protection for the lung [50,51]. The K_m_ and V_max_ for synthetic substrate tosyl-Gly-Pro-Lys-paranitroanilide (Chromozym PL) were 319 mM and 1.33 mM/min, respectively [38]. The K_m_ and V_max_ for Val-Leu-Lys-paranitroanilide were 727 mM and 0.74 mM/min, respectively.

### 2.4. Regulation of Piv Expression

The production of protease by strain PA103 was found to be regulated by a protein designated as the virulence factor regulator (Vfr) that is homologous to the cyclic AMP receptor protein (CAP or CRP) of *E. coli* [52,53]. To activate the upregulation of virulence genes by Vfr, there are two adenyl cyclases that produce cAMP; to prevent over-production of the virulence genes controlled by Vfr, cAMP phosphodiesterase (CpdA) degrades cAMP [54]. High concentrations of cAMP cause Vfr to induce expression of CpdA, which lowers cAMP. Thus, Vfr can upregulate and, also when needed, down regulate the expression of virulence factors [54]. The production of PIV is also regulated by quorum sensing. The quorum sensing regulator LasI is active in controlling the production of pyoverdine and a mutation in this regulator caused a 2-fold reduction in pyoverdine production and a similar or larger reduction in PIV production [55,56,57]. A mutation in the gene for LasI prevented the production of PIV such that no PIV activity could be detected in the culture supernatant [57]. Pyoverdine is a siderophore and when it combines with iron, forming ferripyoverdine, the complex binds to a membrane spanning protein, FpvR, that regulates the sigma factor, PvdS, which up-regulates pyoverdine, exotoxin A, and PIV [58,59]. Involved in this regulatory process is the iron uptake regulator (Fur) that controls the expression of *pvdS*, the sigma factor that is needed for PIV production [60]. During an infection, the concentration of iron in tissues is low and the cleavage of lactoferrin and transferrin helps supply iron to the infecting bacteria [61]. PIV is able to cleave both of these iron-containing proteins and this could aid a developing infection [45]. A high concentration of iron suppresses production of PvdS and, as a result, also the genes that PvdS up-regulates, such as *piv*. Conversely, conditions with restricted amounts of iron favor production of PvdS and the genes that this sigma factor regulates [60]. Biofilms are thought to have limited amounts of iron as compared to most environments in which planktonic bacteria grow, and this is thought to explain the nearly thirty-nine-fold increase in PIV production in a biofilm as compared to cultures of planktonic bacteria [35]. LasR is another quorum sensing regulator that controls PIV production and LasR is influenced by both the amount of iron and oxygen available to the bacteria [62]. Another regulatory factor, which can also influence the production of PIV by regulating the availability of the PvdS sigma factor, is transcription factor CysB. CysB is upregulated by sulfur starvation and is able to bind to the promoter of PvdS, upregulating its expression [63]. Thus, CysB provides a response that increases the expression of virulence genes dependent on PvdS, including *piv*. 

There are additional factors that increase the production of PIV, mainly by mechanisms that are not yet fully elucidated. Calcium and magnesium have been described as inducers of PIV production and the calcium content of a cornea was found to increase when infected with *P. aeruginosa* [64]. Calcium was also found to enhance protease production of *P. aeruginosa* in biofilms by affecting the biofilm structure and enhancing expression of a variety of products [65]. Conditions of growth, such as a decrease in the temperature of incubation and the degree of cell aggregation, can also increase PIV production [66,67]. Reduced amounts of phosphate create a response in *P. aeruginosa* that results in increased pyoverdine, PIV, and exotoxin A; increased phosphate can reverse the increased production of these virulence factors [68,69]. Colonization of the mouse gut, followed by lethal sepsis, occurred when phosphate concentration was low and could be prevented by oral administration of phosphate [68,70]. When the bacteria swarm, there is a 5.2-fold increase in protease IV gene expression [71].

Decreases in PIV production have also been demonstrated to occur in response to specific conditions or reagents. As mentioned above, high phosphate concentrations reduce production of PIV [68,69,70]. A quorum sensing inhibitor, iberin, was found to reduce expression of virulence factors, including PIV [72]. Also, the anti-fungal drug flucytosine was found to inhibit the production of PvdS, the sigma factor needed for PIV and pyoverdine production; this inhibition reduced the production of PIV by at least 5-fold [73]. The effectiveness of flucytosine was demonstrated in a mouse model of pneumonia in which survival afforded by flucytosine was increased from about 20% to about 90%. Also causing a decrease in PIV production is a mutation in one of the lectins produced by *P. aeruginosa*, LecB. A mutant deficient in LecB caused a reduction in type IV pili production and also reduced amounts of PIV [74]. The LecB-deficient mutant is thought to affect type II secretion pathway that is the route for PIV secretion. 

## 3. Virulence of PIV 

### 3.1. Biological Activity of PIV

Perhaps the most impressive attribute of protease IV as a virulence factor is its ability to augment bacterial infection without stimulating a typical immune response to itself as a foreign protein. PIV has been shown to contribute to bacterial virulence in plants, insects, rabbit and mouse corneas, and the mouse and human lung [28,38,40,43,50,51,75,76,77,78,79,80]. Additionally, there is evidence that PIV is active in human lung infections [50]. Despite this strong association with virulence, PIV has been found to be a weak immunogen [81]. Antibody was not produced in rabbits following repeated injections of the active or heat-inactivated PIV molecule. Antibody was obtained in response to multiple administrations of a precipitated recombinant protein (i.e., 6-histidine tagged) consisting of the mature protease region that was not folded into an active protease. The resulting antibody reacted at a high titer with this immunogen and at an approximately 5-fold lower titer with the active PIV molecule [81]. However, this antibody was not able to neutralize the protease activity [81]. Antibody to PIV was detected in the sera of cystic fibrosis patients who had been chronically infected with *P. aeruginosa* [82]. Apparently, prolonged exposure of the immune system to PIV can induce a humoral immune response. The ability of such antibody to neutralize PIV activity is unknown. The role of PIV in virulence in animals and plants is summarized in Table 2.

### 3.2. Infection of Plants 

The application of *P. aeruginosa* to a plant leaf can result in an infection characterized by soft leaf rot [85]. A model system for studying the pathogenesis of *P. aeruginosa* on plants has been established using the plant *Arabidopsis*, which is commonly called thale cress and is a member of the cabbage family of plants [77]. The bacteria have been found to induce a plant immune pathway that is homologous to the mammalian induction of innate immunity involving an oxidative response. Active PIV, but not heat-inactivated or inactive mutant PIV, was able to induce this immune response system that involves the activation of a series of proteins including Gα, Gβ, and Gγ, followed by activation of MAP kinases and RACK1 [77]. Thus, the plant response system activated by PIV is analogous to that of mammalian cells. Cheng et al. concluded that an evolutionary and physiological interpretation of their findings is that plants evolved a novel surveillance system to recognize and respond to pathogen-encoded proteases that disrupt host homeostasis via their proteolytic activity. The host response was activated by the degradation of host protein mediated by PIV (and possibly other bacterial proteases), but not by the mammalian protease, trypsin [77].

### 3.3. Infection of Insects

*P. aeruginosas* is a pathogen active against a variety of insect species and the role of PIV as a virulence factor has been studied in model systems using mealworms (*Tenebrio molitor*) or the wax moth (*Galleria mellonella*) [78]. In *T. molitor*, PIV was found to degrade the zymogens of spätzle processing enzyme (SPE) and SPE-activating enzyme (SAE) [79]. These two enzymes activate spätzle, which is a ligand for Toll-like receptor activity in an innate immune response. The Toll-like receptor is able to signal the production of antimicrobial peptides that protect the host from the bacterial infection [79]. These results suggest that PIV provides *P. aeruginosa* with a sophisticated way to escape the immune attack of the host by interfering with the production of antimicrobial peptides. In *G. mellonella*, the infection was present in the hemolymph and reduced the activity of granulocytes and plasmatocytes [78]. PIV is also able to degrade apolipophorin III in the insect’s haemolymph and this destruction removes a carrier of lipid as well as a pattern recognition molecule able to increase the antibacterial activity of the haemolymph [75,86]. Apolipophorin III provides antibacterial activity to the haemolymph by enhancing lysozyme production and increasing phagocytosis by haemocytes. PIV is thought to be the bacterial factor that reduces the amount of apolipophorin III in the haemolymph during a *P. aeruginosa* infection [75,76]. 

### 3.4. Infection of Mammalian Corneas 

The most commonly recovered bacteria from corneal scrapings in contact lens-related microbial keratitis include *Pseudomonas*, *Serratia*, and *Staphylococcus* [87]. Howe and Iglewski [18] reported that strain PA103 produced alkaline protease and a mutation in the gene for this protease caused a loss in virulence for the mouse cornea that could be overcome by the addition of exogenous protease at the time of infection. Twining et al. [88] reported that *P. aeruginosa* strain PA103 produced proteinases other than elastase and alkaline protease. Twining et al. also found that the *Pseudomonas* proteases, including proteinase other than elastase or alkaline protease, could cleave corneal proteases, causing their activation or degradation. O’Callaghan et al. [28] found that strain PA103 produced PIV and that the mutant PA103-AP1 produced only 5% of the parental amount of PIV. PA103-AP1 was significantly less virulent in models of keratitis in the mouse or rabbit eye. Another mutant PA103-29 found to have markedly reduced PIV production was also found to have a marked reduction in corneal virulence in both the rabbit and mouse eye [83]. The injection into the rabbit cornea of a PIV-deficient mutant caused minimal pathology whereas the infection with this strain supplemented with exogenous active PIV, but not heat-inactivated PIV, caused a much more virulent infection [84]. The same study showed that highly active PIV injected into the rabbit cornea can cause epithelial damage. A strain of *P. aeruginosa* (Paer1) isolated from a human case of contact-lens-induced-red-eye (CLARE) was minimally virulent in a mouse eye even though it was able to produce alkaline protease, but not elastase A, elastase B, and PIV [89]. Restoration of its ability to produce elastase B did not increase the virulence of the CLARE isolate suggesting that PIV and elastase A, but not elastase B or alkaline protease, could be key virulence factors for human eyes. As noted above, strain PA103 and its derived mutant strains lack elastase B production yet retain full corneal virulence [40,47]. 

Analysis of *Pseudomonas* isolates showed that there are two types of protease producers; type one organisms produce PIV, LasB, and alkaline protease whereas type two producers make PIV, modified LasB (or PASP), and alkaline protease [90]. Type one protease production was common among non-contact lens keratitis isolates and Type II protease producers were common among contact lens associated keratitis isolates [90]. In a separate study, the isolates producing type two proteases were found to harbor the *exoU* gene whereas isolates with the type one proteases harbored the *exoS* gene [91]. 

A PIV mutant was analyzed in a rabbit model of keratitis and found to grow as well as its isogenic parent strain but was significantly less virulent [40]. The virulence of the PIV-deficient mutant could be restored by complementing the mutated *piv* gene with a functional *piv* gene on a plasmid, a finding that proves the role of PIV as a corneal virulence factor. Another form of evidence for the role of PIV in corneal virulence was based on the use of a strain of *P. putida* that lacked secreted protease activity [42]. This *P. putida* strain lacked virulence in the rabbit model of keratitis, but acquired a significant amount of virulence when it was genetically engineered to produce and secrete active PIV from a plasmid-borne copy of the *piv* gene. An interesting finding was that elastase B, but not alkaline protease, expressed by *P. putida* produced significantly more corneal damage than the *P. putida* protease-free parent [42]. 

Conibear et al. [43] found that human ocular isolates had the *piv* gene, but production of the enzyme varied and was controlled by the quorum sensing system. Strains with low production of the quorum sensing molecules (LasR and LasI) produced little to no protease IV; complementation of the quorum sensing system resulted in increased PIV production. In a study of a strain isolated from a corneal ulcer (strain KEI 1025) and a strain isolated from CLARE (strain Paer1), the KEI 1025 strain was found to produce a virulent keratitis infection of mice whereas the Paer1 produced a mild transient infection [89]. When tested in vitro, the Paer1 strain lacked elastase A, LasB, and PIV, but the virulent KEI 1025 strain produced these three proteases. Restoration of LasB production in Paer1 did not provide virulence in the mouse model of keratitis, a finding that supports the concept of LasB failure to mediate significant virulence in experimental keratitis [89]. In a more recent study, the amount of PIV in a *Pseudomonas* infection of caprine corneas (ex vivo) increased during the course of the infection and that the increase in PIV was greater than the increase of other molecules analyzed [92].

### 3.5. Protease IV in the Respiratory Tract 

#### 3.5.1. Surfactant Proteins 

The respiratory system has mucous membranes that in some ways resemble the anterior ocular surface and the two sites are subject to severe infection with *P. aeruginosa*. One feature of these sites is the presence of surfactant proteins that can aggregate and opsonize bacteria [50]. The ability of *P. aeruginosa* to cause respiratory dysfunction, often leading to the death of cystic fibrosis patients, and be a leading pathogen in nosocomial pneumonia requires that it overcome or avoid the protective host defenses of the respiratory tract, including the surfactant proteins. Purified PIV was found to digest surfactants A, D, and B in cell-free bronchoalveolar lavage fluid [50]. Such digestion of surfactant proteins was inhibited by TLCK, an inhibitor of PIV [38]. These findings are consistent with PIV, contributing to the development of a respiratory tract infection. 

#### 3.5.2. Rat Lung Infection

When the wild-type and a PIV-deficient mutant were compared in an agar bead model of pneumonia in rats, the wild-type bacteria, by seven days post-infection, achieved a 14-fold larger bacterial population (CFU) than the same strain rendered deficient in PIV [45]. This major increase in the number of the PIV-producing (wild-type) bacteria relative to the PIV-deficient bacteria occurred despite a 1.5-fold larger population of the PIV-deficient bacteria in the inoculum [45]. 

#### 3.5.3. Human Cytokine IL-22 

PIV was analyzed as a molecule active in human ventilator-associated pneumonia [51]. The lung is thought to be protected by IL-22 that signals the release of human beta-defensin 2, an antimicrobial peptide active on *Pseudomonas* [51]. IL-22 is unusual among interleukins because it targets cells in the body’s barriers, including the respiratory epithelial cells. In vitro tests of purified preparations of four key *P. aeruginosa* proteases (i.e., LasB, AP, PASP, and PIV) showed that only PIV could digest recombinant IL-22 [51]. Natural protease inhibitors in the lung were unable to inhibit PIV and sputum from patients with *P. aeruginosa* pneumonia contained active PIV. Recombinant IL-22 added to sputum from patients with *Pseudomonas* pneumonia was rapidly digested whereas sputum from patients with other forms of pneumonia failed to digest recombinant IL-22 [51]. Thus, the activity of PIV seems to provide *P. aeruginosa* with a means to destroy IL-22 and prevent the signal needed to induce the release of protective human beta-defensin 2. The destruction of IL-22 by PIV to prevent antimicrobial peptide release is analogous to the role of PIV in destroying apolipophorin III in the insect haemolymph to prevent insect antimicrobial peptide release [75]. The association between PIV and human lung infections was also found by the study of strains isolated from patients with cystic fibrosis. Smith et al. found that 95% of the *P. aeruginosa* isolates from cystic fibrosis patients produced PIV [93].

#### 3.5.4. Augmenting Pneumococcal Lung and Systemic Infection

The finding of PIV cleavage of IL-22 in sputum from *Pseudomonas* pneumonia patients suggested that PIV could render the lung susceptible to severe infection by not only *P. aeruginosa* but also other pathogens. Thus, a study was initiated to determine if PIV or co-infection with *P. aeruginosa* producing PIV could augment the virulence of a low virulence strain of *Streptococcus pneumoniae* (i.e., pneumococcus). The interaction between PIV and pneumococcus in the lung relates to the finding that a significant percentage of patients with pneumonia can have two bacteria infecting their lung, including pneumococcus and *P. aeruginosa* [94]. When pneumococcus and *P. aeruginosa* concurrently infect a lung, the pleural effusions and cavity formation are significantly more frequent than for a single pathogen cause of pneumonia [95]. 

The main pneumococcal strain (i.e., EF3030) involved in the study with PIV has a limited ability to replicate in the mouse lung, and is inefficient in invading the blood stream following its inoculation into the trachea [80]. When this pneumococcal strain (10^6^ colony forming units, CFU) was inoculated with *Pseudomonas* elastase B (LasB), or heat-inactivated PIV (i.e., non-enzymatic), after two days there were 4 to 5 logs CFU of pneumococci in the lung and approximately 1 log CFU in the blood; none of the mice died from these infections. However, when these pneumococci were inoculated with active PIV (10 µg), by two days post-infection the mouse lung contained more than 7 logs CFU of pneumococci and the mouse blood contained 5 logs CFU; 100% of the mice died from this infection [80]. The mice inoculated with EF3030 and active PIV contained by day 2 post-infection less than 50 pg/mL of IL-22 in the lung homogenate whereas lungs infected with EF3030 alone, EF3030 plus heat-inactivated PIV, or EF3030 plus LasB contained approximately three-fold more IL-22 (i.e., 120 to 170 pg/mL). The stimulation of pneumococcal infection by purified PIV could be achieved also by co-infecting the lung with this low virulence pneumococcus and wild-type *P. aeruginosa* (strain PA103-29) [80]. However, the co-infection of this pneumococcus with the PIV-deficient mutant of PA103-29 failed to produce the virulent infection obtained when the wild-type PA103-29 was used for the co-infection. These experiments implicate PIV as a highly active virulence factor able to significantly limit the host defenses of the lung.

## 4. PASP: A Second Ocular Virulence Factor

The *Pseudomonas aeruginosa* small protease (PASP) was first described by Marquart et al. as a product of strain PA103 [29]. PASP was detected on zymograms as an 80 kDa protease and was distinguished from modified elastase, which also appears on zymograms as an 80 kDa protease, by its failure to react with antibody to elastase. The enzyme has a molecular mass of 18.5 kDa and the *Pasp* gene of strain PAO1 is located at PA0423 position on the *P. aeruginosa* chromosome and is >99% identical to that of strain PAO1 [29]. The *Pasp* gene was detected by PCR reactions in all strains of *P. aeruginosa* tested (*n* = 25) and polyclonal antibody to recombinant PASP detected native PASP protein in the supernatant of all strains tested [30]. PASP was found to cleave collagens I and IV and to be inactivated by TLCK, a serine protease inhibitor [30]. 

The cleavage of collagens is an important finding relative to corneal infections because PIV has relatively little activity on collagens implying that PASP, not PIV, has the function of producing corneal epithelial erosions [30]. Injection of PASP into the rabbit cornea caused both corneal erosions and erosion of the corneal stroma [29,30]. The cornea injected with PASP also showed neutrophil infiltration, a reaction observed by both slit lamp examination and histological analysis [30]. In addition to cleaving collagens, PASP was found to cleave complement component C3, fibrinogen, and anti-microbial peptide LL37 [31]. PASP was also found to cleave multiple proteins of the rabbit tear film. A PASP-deficient mutant of PA103-29 was found to grow in the rabbit cornea as well as its isogenic parent and its complemented (rescue) strains, but its virulence was significantly lower than the two strains able to produce PASP [31]. The reduced virulence of the PASP-deficient mutant relative to the isogenic parent and rescued strain clearly establish PASP as a toxic corneal virulence factor. 

The studies of the substrate specificity of PASP showed that PASP cleaves at lysine or arginine [32]. Site-directed mutagenesis of the *Pasp* gene determined that residues Asp-29, His-34, and Ser-47 comprise the catalytic triad of this serine protease. Substitution of alanine for any of these residues eliminated protease activity. Injection into the rabbit cornea of PASP with alanine substituted for serine at position 47 resulted in a slit lamp examination (SLE) score of near 0.0 (i.e., a normal eye) whereas injection of PASP with alanine substituted for a serine outside of the catalytic triad (i.e., at position 59) produced a SLE score of about 9.0 [32]. This finding emphasizes the critical importance for protease activity to obtain corneal toxicity. 

When the model of PASP, based on its structural similarity to the *E. coli* protein YceI, was analyzed for the location of the catalytic triad, there was a significant distance between the location of Asp-29 and His-34 relative to that of Ser-47 [32]. Such a distance indicated that these amino acid residues on a single protein could not function as a catalytic triad; however, when two molecules of PASP are oriented as a dimer, the Ser-47 on one monomer is proximal to the Asp-29 and His-34 of the other monomer. This model was confirmed by the finding that the protease activity of recombinant PASP, in contrast to native PASP migrating at 80 kDa, observed on zymograms appeared at the molecular size of a dimer (~40 kDa) and not at that of the monomer [32]. Furthermore, Western blot analysis demonstrated the presence of dimers and monomers in preparations of purified recombinant PASP and its site-directed mutants of the catalytic residues [32]. 

Associations have been made between human lung infections and the production of PASP and PIV. Strains of *P. aeruginosa* isolated from patients with cystic fibrosis have been found to produce more of these two proteases than other strains that were tested, including the prototype PAO1 strain [82,96,97]. These lung infections involve extensive amounts of bacterial biofilms and such biofilms have been related to ocular infections and to the up-regulation of PASP and PIV production [35,98]. Biofilms produce thirty-nine-fold more PIV and five-fold more PASP than planktonic cultures [35]. Patients with cystic fibrosis in Australia were found to produce antibody to PASP, indicating that PASP is produced in vivo [82]. 

## 5. Conclusions

PIV has the ability to cleave a wide variety of proteins, including many involved in host defense, thus favoring the development of an infection. This protease contains only weak immunogenic sites and antibody specific for PIV is very uncommon and neutralizing antibody to PIV has not been reported. The diverse role of PIV as a virulence factor has been described in models of infection of plants, insects, mammalian eyes, and mammalian lungs, including human lungs. Evidence shows that PIV, by weakening the host defenses, can enhance an infection by other bacteria. Because PIV plays an important role in human infections, inhibitors of its activity or antibody able to neutralize its activity could become an important adjunct therapy for preventing infections or limiting the tissue damage of an infection. The full range of host proteins cleaved by PIV and the effect of PIV on the surface of *P. aeruginosa* and other bacteria in the body’s microbiome are essentially unknown. 

The one aspect of a corneal infection that may not extensively involve PIV is the erosion of the collagen-rich cornea. Such erosion appears to be more readily mediated by PASP, a serine protease with the ability to cleave collagens as well as multiple host defense proteins. The potency of PIV on a spectrum of host defense proteins complemented by the collagen cleaving ability of PASP creates a challenge to the cornea during *Pseudomonas* keratitis. Additionally, PASP, like PIV, has been recognized as a virulence factor in experimental keratitis and found to be a common product of *Pseudomonas* during the infection of the human respiratory tract.

## Figures and Tables

**Table 1:** Summary of *Pseudomonas aeruginosa* secreted proteases.

Name	Abbreviation	Type	Comment	Reference
Elastase A	LasA	Metalloprotease	Staphylolytic protease	[25]
Elastase B	LasB	Metalloprotease	Potent enzyme and virulence related	[25]
Alkaline Protease	AP	Metalloprotease	Broad spectrum protease and virulence related	[26]
Protease IV	PIV (Ps-1; PrpL)	Serine Protease	Lysine specific and important virulence factor	[27,28]
*Pseudomonas aeruginosa* Small Protease	PASP	Serine Protease	Cleaves collagen and other proteins, virulence factor	[29,30,31,32]
Large Protease	LepA	Serine Protease	Activates inflammation and virulence factor	[33]
Autotransporter Protein	EprS	Serine Protease	Activates inflammation	[34]
Metzincin Protease	Mep72	Metalloprotease	Biofilm secreted protein	[35]
ATCC27853 Protease	/	Metalloprotease	Stable in organic solvents	[36]

**Table 2:** Summary of the virulence functions of protease IV.

Type of Host	Host Studied	Effect on Host	References
Plant	*Arabidopsis* (thale cress, a cabbage)	Disruption of normal homeostatis and induction of a defensive host response	[77]
Insect	*Tenebrio molitor* (mealworms) or *Galleria mellonella* (wax moth)	Bacteremia protected by PIV-mediated killing of defensive cells. Also, destruction of apolipophorin III to reduce phagocytosis of bacteria	[75,76,78,79]
Mammals	Rabbit corneal infection	Destruction of host defensive proteins and possibly collagens and induction of inflammation	[38,39,40,41,42,64,81,83,84]
	Rodent respiratory tract	Wild-type *P. aeruginosa* in agar beads had more colony forming units that a mutant deficient in PIV.	[45]
	Rodent respiratory tract	Destruction of host defense proteins (surfactants A, D, and B) that opsonize the bacteria. Also, enhance bacteria (CFU) in lung more than PIV-lacking mutant	[50]
	Mouse lung	PIV enhanced pneumococcal lung infection and bacteremia by destroying IL-22 in the lung.	[80]
	Human lung	Destroy IL-22 in human lung fluids including sputum of patients with *Pseudomonas* pneumonia	[51]
